# Development and Comparability of Internalizing and Externalizing Symptom Spectra From Adolescence to Young Adulthood

**DOI:** 10.1002/mpr.70055

**Published:** 2026-01-21

**Authors:** Vera Birgel, Michael Rapp, Mira Tschorn, Heike Hölling, Caroline Cohrdes

**Affiliations:** ^1^ Department of Epidemiology and Health Monitoring Robert Koch Institute Berlin Germany; ^2^ German Center for Mental Health (DZPG) Berlin Germany; ^3^ Department of Social and Preventive Medicine University of Potsdam Potsdam Germany

**Keywords:** externalizing symptoms, HiTOP, internalizing symptoms, measurement comparability, transition to adulthood

## Abstract

**Objectives:**

This study examines the continuity and comparability of internalizing and externalizing symptom spectra from adolescence to young adulthood, addressing measurement challenges across developmental stages. Leveraging the Hierarchical Taxonomy of Psychopathology (HiTOP) framework, it explores whether symptom spectra in adolescence predict corresponding symptoms in young adulthood.

**Methods:**

Data were drawn from JEPSY, a follow‐up of the national KiGGS cohort (*N* = 2172, age 18–26). Adolescent internalizing and externalizing symptoms were assessed via the Strengths and Difficulties Questionnaire (SDQ) and substance use items. Adult outcomes included the Patient Health Questionnaire (PHQ‐4), DSM‐5 Cross‐Cutting Symptom Measure (DSM‐5 CC), and Personality Inventory for DSM‐5 (PID‐5). Factor analyses assessed structural consistency, and robust regression examined associations between adolescent and adult symptom spectra over 7–10 years.

**Results:**

A four‐factor model best captured the SDQ structure. In young adulthood, three spectra emerged: internalizing symptoms, externalizing traits, and substance use. Adolescent emotional and peer problems predicted internalizing symptoms in adulthood. Conduct problems and hyperactivity predicted externalizing traits. Substance use was associated with hyperactivity, smoking, and risky drinking—but negatively with peer problems.

**Conclusion:**

The findings support the continuity of broad psychopathological spectra and demonstrate that harmonized approaches can bridge measurement gaps and enhance longitudinal comparability.

## 1 Introduction

Recent studies indicate a notable rise in mental health problems and disorders among children and adolescents (Askari et al. 2023; Patalay and Hardman 2019; Wiens et al. 2020). Findings from a representative sample of U.S. students suggest that internalizing symptoms (e.g., anxiety, depression) have increased significantly since 2010, affecting both boys and girls. In contrast, externalizing symptoms (e.g., aggression, attention difficulties), have declined, particularly among boys. Despite these opposing trends, a positive correlation persists between internalizing and externalizing symptoms, suggesting that adolescents with high internalizing symptoms are also more likely to exhibit externalizing symptoms (Askari et al. 2023).

These findings align with growing evidence that internalizing and externalizing problems often co‐occur and exist on a continuum, rather than as distinct categories, underscoring the need for an integrated perspective on psychopathology (Achenbach et al. 2016). Additionally, epidemiological research shows that mental health problems in youth are both prevalent and persistent, often continuing into adulthood (Collishaw 2015). Understanding how psychopathology evolves across developmental stages is crucial for identifying risk factors, protective mechanisms, and critical periods for intervention. Longitudinal cohort studies spanning childhood to young adulthood provide valuable insights into mental health trajectories, underlying influences, and gaps in healthcare provision (Bogdan et al. 2023; Holz et al. 2021). This developmental period is particularly important, as early‐life experiences play a key role in shaping mental health trajectories and influencing the emergence and manifestation of psychopathology in later life (Moffitt et al. 2006; Shonkoff et al. 2012).

Since the 1970s, population‐based longitudinal studies have suggested moderate stability in psychopathology from adolescence into adulthood, with externalizing symptoms showing greater persistence than internalizing ones (Caspi et al. 1996; Ferdinand and Verhulst 1995). However, few studies have extended their focus into adulthood, which is essential for understanding the long‐term trajectories of psychopathology (Caspi et al. 1996; Ferdinand and Verhulst 1995; Hofstra et al. 2000; Reef et al. 2009).

Beyond these spectrum‐level links, work on the general factor of psychopathology (*p*) has demonstrated substantial shared variance across common symptoms and disorders, helping explain robust comorbidity both cross‐sectionally and longitudinally (Caspi et al. 2024). Developmentally, *p* can already be identified in childhood and shows relative stability across late childhood and adolescence, co‐existing with meaningful spectrum‐specific variance (Murray et al. 2016). Recent adolescent studies further connect *p* with maladaptive personality traits (Benzi et al. 2025, 2024), reinforcing the value of modeling both general and domain‐specific liabilities.

A significant challenge in studying psychopathology across developmental stages lies in the inconsistency of assessment instruments. While tools such as the Child Behavior Checklist (CBCL) and its adult counterpart, the Young Adult Behavior Checklist (YABCL), are designed to measure behaviors consistently from childhood through adulthood, such comprehensive instruments are rare. In large‐scale population studies, their use is often limited by licensing costs, survey length, and resource constraints. The lack of comparable measures across age groups complicates efforts to trace developmental trajectories. Furthermore, transitions between informants (e.g., from parent and teacher reports in childhood to self‐reports in adulthood) and the use of age‐specific methodologies introduce additional complexities, including potential biases, informant discrepancies, and limited convergence across methods. These challenges underscore the importance of harmonized, developmentally informed approaches in longitudinal psychopathology research (Achenbach et al. 2016; Wilson and Olino 2021). In parallel, current debates in nosology advocate transdiagnostic specifiers to better capture shared liabilities and developmental course across traditional categories, especially in child and adolescent psychiatry (Peña et al. 2024).

Additionally, many studies have focused primarily on clinical samples or discrete classifications of mental disorders, such as depression (Fleming et al. 1993) or anxiety (Roza et al. 2003). These approaches, while valuable, miss the broader interconnected dimensions of internalizing and externalizing behaviors, which are crucial for understanding general patterns of mental health and identifying universal strategies for intervention and prevention strategies (Wilson and Olino 2021).

The Hierarchical Taxonomy of Psychopathology (HiTOP) model provides an alternative to traditional diagnostic systems like the DSM and ICD, which categorize mental health disorders as discrete entities and put emphasis on neurobiology. Instead, HiTOP conceptualizes psychopathology as a continuum from normality to pathology, providing a more nuanced understanding of how symptoms interact and co‐occur across different spectra. By organizing psychopathology hierarchically—from specific symptom clusters to broad spectra like internalizing and externalizing—HiTOP addresses challenges regarding the complexity of mental disorders, including issues of diagnostic instability and comorbidity (Kotov et al. 2017). Embedding HiTOP within a hierarchical view that acknowledges a potential general liability (*p*) above spectra clarifies why spectra covary and how spectrum‐specific variance remains informative (Caspi et al. 2024; Krueger et al. 2021; Murray et al. 2016). In the context of this study, HiTOP underscores the importance of considering broad dimensions such as internalizing and externalizing symptoms, allowing for a meaningful comparison of outcomes across various developmental stages.

The utility of the HiTOP model, however, relies on thorough empirical validation through population‐based research. While widely used tools such as the DSM‐5 Cross‐Cutting Symptom Measure (DSM‐5‐CC), the Patient Health Questionnaire‐4 (PHQ‐4), and the Personality Inventory for DSM‐5 (PID‐5) capture important aspects of psychopathology, their ability to assess broader dimensions like internalizing and externalizing symptoms across developmental stages remains underexplored. This limitation reduces their applicability for dimensional approaches that aim to move beyond categorical diagnoses and reflect the hierarchical structure of psychopathology.

These measurement challenges become become particularly relevant when considering nomological networks, which describe relationships between different constructs and their underlying structures. Numerous risk and outcome variables (e.g., early life stress, social support, academic achievement, quality of life) are theorized to relate to higher‐order dimensions of psychopathology rather than to specific disorders or symptom clusters. Accordingly, it is essential to evaluate whether commonly employed instruments, such as the PHQ‐4 and DSM‐5‐CC, validly reflect internalizing and externalizing dimensions as conceptualized within the HiTOP framework. While the PID‐5 is already listed as a HiTOP‐friendly measure, other widely used instruments such as the PHQ‐4 and DSM‐5 Cross‐Cutting Symptom Measure have not yet undergone systematic validation in this context. Given that the current HiTOP recommendations are not exhaustive, examining the structural alignment of such tools remains essential. Positioning these measures within the HiTOP framework allows for testing their structural validity and comparability across developmental stages. Moreover, such analyses can help determine whether these instruments are appropriate for bridging measurement discontinuities that frequently arise from changes in assessment tools across age groups—especially relevant given the high prevalence and persistence of internalizing and externalizing psychopathology. Particularly in longitudinal and population‐based studies, ensuring measurement continuity across age groups is essential for reliably tracking the progression and intensity of psychopathological symptoms over time.

## 2 Aim of the Study

This study builds on data from the JEPSY study, the follow‐up of a representative nationwide health survey of children and adolescents (KiGGS). In KiGGS, child and adolescent mental health problems were assessed using the Strengths and Difficulties Questionnaire (SDQ), which allows differentiation between internalizing and externalizing domains. In the JEPSY follow‐up, internalizing and externalizing psychopathological symptoms were reassessed using the PHQ‐4, DSM‐5‐CC, and the Personality Inventory for DSM‐5 (PID‐5). The selection of measures was guided by an expert commission within the framework of the German Center for Mental Health to establish a minimum dataset (MDS) for longitudinal mental health monitoring (Tschorn 2025).

The primary objective was to determine whether these symptom spectra, as conceptualized by the Hierarchical Taxonomy of Psychopathology (HiTOP) model, can be reliably measured despite differences in assessment tools used across developmental stages. Specifically, we aimed to investigate the extent to which internalizing and externalizing symptom spectra assessed in adolescence align with those measured in young adulthood via different measurement instruments. Thus, we aimed to evaluate whether these instruments provide a valid methodological bridge for studying psychopathology across developmental stages. As a secondary aim, we examined whether adolescent internalizing and externalizing symptom patterns predict corresponding dimensions in young adulthood, thereby capturing longitudinal associations across the transition to adulthood.

## 3 Methods

### 3.1 Participants and Procedure

The JEPSY study is a longitudinal online follow‐up of participants from the German Health Interview and Examination Survey for Children and Adolescents (KiGGS), conducted by the German national health institute (Robert Koch Institute; RKI). KiGGS is a nationally representative cross‐sectional study assessing the health of children and adolescents aged 0–17 years (Baseline 2003–2006, first wave 2007‐2011, second wave 2014‐2017) as well as an additional longitudinal cohort following participants into early adulthood (Hoffmann et al. 2018; Mauz et al. 2020).

For JEPSY, 11,737 KiGGS Wave 2 participants aged 16–25 in April 2024 were invited to participate. Eligibility criteria included: (i) age between 16 and 25 at the start of data collection, (ii) prior consent for recontact, and (iii) no known factors precluding participation (e.g., permanent relocation abroad) (Lange et al. 2025).

A total of 3063 individuals completed the JEPSY questionnaire. For regression analyses, only participants who were at least 11 years old at the time of KiGGS (when self‐reports on the SDQ were available) were included. Among eligible participants, SDQ data completeness exceeded 90% across subscales, with missing rates ranging from 5.5% (emotional symptoms) to 6.6% (peer problems). Mental health measures in young adulthood (PHQ‐4, PID‐5, DSM‐5‐CC) had completion rates of 99.9% or higher.

Missing data patterns were assessed using Little's MCAR test, which suggested that data for both adolescence and adulthood are likely Missing at Random. This was further supported by a non‐significant Anderson‐Darling test, indicating no deviation from multivariate normality. Given the complexities of imputing mental health data and potential biases, participants with missing values in any mental health outcome were excluded casewise from regression analysis.

After applying inclusion criteria and accounting for missing data, the final analytical sample comprised 2172 participants aged 18–26 with complete mental health data from both developmental stages. To validate factor structures and ensure comparability, the derived factor solution was cross‐validated in the Minimum Dataset (MDS) of the German Center for Mental Health, an independent representative sample using the same psychometric instruments (Tschorn 2025) (see Appendix I for study description).

### 3.2 Measures

#### 3.2.1 Early Adolescence

Mental health problems in adolescence were assessed using the Strengths and Difficulties Questionnaire (SDQ), a widely used screening tool for children and adolescents (4–17 years). The SDQ comprises 25 items across five subscales: emotional symptoms, conduct problems, hyperactivity/inattention, peer relationship problems, and prosocial behavior. Each item is rated on a three‐point scale. For this study, four of these subscales were utilized to derive internalizing and externalizing spectra except the prosocial behavior scale, as suggested before(Goodman 2001). The internalizing dimension included emotional problems and problems with peers. The externalizing dimension captured conduct problems and hyperactivity.

Substance use was assessed via smoking frequency and the Alcohol Use Disorders Identification Test‐Consumption (AUDIT‐C), classifying participants as non‐drinkers, moderate drinkers, or at‐risk drinkers. Socioeconomic status (SES) was measured using a standardized composite score based on parental education, occupational status, and household income (range: 1–7), with higher values indicating higher SES (Lampert et al. 2014).

#### 3.2.2 Young Adulthood

At the JEPSY follow‐up, mental health was reassessed using multiple validated instruments, several of which aligned with HiTOP.

The PHQ‐4 was applied to measure symptoms of anxiety and depression through its two subscales, the PHQ‐2 and GAD‐2, which separately capture depressive and anxiety symptomatology. Each item is rated on a four‐point Likert scale (0 = “Not at all” to 3 = “Nearly every day”).

A subset of the DSM‐5‐CC was used to assess internalizing and externalizing symptoms, including depression, anxiety, substance use, and sleep problems. Items were rated on a three‐point scale (0 = “Not at all” to 2 = “Very much”).

A subset of the PID‐5 was employed to assess maladaptive personality traits, particularly those relevant to the externalizing dimension. Personality traits assessed in this study include risk taking, irresponsibility, deceitfulness, eccentricity, and impulsivity. Items were rated on a five‐point Likert scale ranging from 0 (“Very false or often false”) to 4 (“Very true or often true”).

Beyond the guidance of the DZPG Minimum Data Set, item selection followed an a priori mapping to the HiTOP spectra to ensure face‐valid coverage of three targets—internalizing symptoms, trait‐like externalizing (disinhibition/antagonism), and substance use—using brief, license‐free items. Selection emphasized developmental appropriateness and clear wording for population surveys, parsimony (minimizing redundancy by retaining one representative indicator per narrow facet where feasible), and cross‐domain comparability of response formats to support harmonization and downstream factor modeling. The complete list of administered items and response formats is shown in Table 1.

**TABLE 1:** (Unweighted) descriptive statistics of psychopathological symptoms and sociodemographic characteristics (*n* = 2172).

Characteristics	*N*	Range		%	*M* (SD)
		Min	Max		
*t*(0) Age 11–17 KiGGS study					
Emotional problems					
Somatic		0	2		0.42 (0.62)
Worries					0.86 (0.74)
Unhappy					0.29 (0.55)
Nervous					0.74 (0.72)
Fears					1.87 (0.72)
Peer problems					
Often alone		0	2		0.68 (0.65)
Prefers adults^a^					0.46 (0.60)
Good friend^a^					0.13 (0.37)
Popular					0.68 (0.59)
Bullied					0.14 (0.41)
Hyperactivity					
Restless		0	2		0.51 (0.64)
Fidgety					0.54 (0.65)
Distractible					0.61 (0.64)
Reflective^a^					1.68 (0.55)
Attentive^a^					2.13 (0.74)
Conduct problems					
Tempers		0	2		0.48 (0.63)
Obedient^a^					0.60 (0.53)
Fights					0.07 (0.26)
Lies					0.17 (0.43)
Steals					0.07 (0.29)
Drinking (Audit‐C)					
Non‐drinker	1361			62.66	
Moderate	592			27.26	
Risky	219			8.94	
Regular smoking					
No	2123			97.74	
Yes	49			2.26	
Parent socioeconomic status					
Occupation		1	7		4.69 (1.38)
Education					5.05 (1.55)
Income					4.53 (1.71)
Age		11	17		13.80 (1.90)
*t*(1) Age 18‐26 JEPSY study					
DSM‐5 CC items					
Alcohol use		0	4		0.83 (0.77)
Tabacco use					0.79 (1.35)
Prescription drug use					0.30 (0.73)
Non‐prescription drug use					0.13 (0.53)
Nervous					1.43 (1.21)
Panic					0.64 (0.97)
Avoiding situations					0.79 (1.11)
Sleep problems					1.63 (1.15)
Anhedonia					1.23 (1.07)
Social anhedonia					1.17 (1.10)
Detachment					1.14 (1.13)
PHQ‐4 Items					
PHQ1		0	3		0.89 (0.81)
PHQ2					0.75 (0.84)
GAD1					1.13 (0.86)
GAD2					0.94 (0.96)
PID 5 Items					
Risk taking		0	3		0.69 (0.76)
Irresponsibility					0.44 (0.66)
Eccentricity					0.95 (0.85)
Deceitfulness					0.50 (0.70)
Impulsivity					0.80 (0.81)
Age		18	26		21.90 (2.00)
Sex					
Female	1483			69.72	
Male	689			30.28	
Education					
No/low	27			12.43	
Middle	363			16.71	
High	1751			80.62	
In education	31			14.27	
Household composition					
Living alone	564			22.89	
Not living alone	1608			77.11	
Health status					
Very good/good	1788			82.23	
Fair/bad/very bad	384			17.77	

Abbreviations: M, Mean; SD, standard deviation.

^a^ Reverse coded items were recoded so that lower values indicate higher symptoms.

As covariates, several sample characteristics were included: sex at birth, age, educational attainment, living situation (alone vs. not), general health status (assessed via the Minimum European Health Module, dichotomized as very good/good vs. fair/bad/very bad) (European Health Expectancy Monitoring Unit 2010), and the subjective social status (SSS), measured using the MacArthur Scale (1 = lowest to 10 = highest perceived social status) (Hoebel et al. 2015).

### 3.3 Data Analysis

The analysis proceeded in two steps: (1) examination of the factorial structure of internalizing and externalizing spectra across adolescence and young adulthood, and (2) regression analyses assessing longitudinal associations between adolescent symptom dimensions and young adult outcomes.

To evaluate the factor structure of the SDQ, a CFA using the Weighted Least Squares Means and Variance adjusted (WLSMV) method was conducted, as this estimator is particularly suitable for ordinal data. While prior research supports the SDQ's ability to distinguish between internalizing and externalizing symptoms, concerns remain regarding its factor structure (Hagquist 2007; Karlsson et al. 2022; Van Roy et al. 2008). Therefore, both a two‐factor model (internalizing vs. externalizing) and a four‐factor model (emotional problems, conduct problems, hyperactivity, and peer problems) were tested. Items that did not load sufficiently onto their theoretically assumed factors were iteratively removed until a stable model was achieved. Model fit was evaluated using multiple indices, with acceptable thresholds set at Root Mean Square Error of Approximation (RMSEA) < 0.06, Comparative Fit Index (CFI), Tucker‐Lewis Index (TLI) ≥ 0.90, and Standardized Root Mean Square Residual (SRMR), with acceptable thresholds set at < 0.08 (Schermelleh‐Engel and Müller 2003).

For young adulthood, an EFA was conducted on PHQ‐4, DSM‐5‐CC, and PID‐5 items to identify latent structures for internalizing and externalizing dimensions. Principal axis factoring with Promax rotation was used, allowing correlated factors. The resulting factor solution was validated using CFA with WLSMV. Additionally, the derived factor structure was cross‐validated using data from a representative German study restricted to the 16–25 age group (*N* = 232, 46.12% male/female) for comparability (see Appendix I for study description). This study was conducted with the objective of establishing and validating a minimum dataset for later use in research conducted by the German Center for Mental Health (Meyer‐Lindenberg et al. 2023).

Following the identification of latent factors, factor scores were computed for internalizing and externalizing symptom dimensions in both adolescence and young adulthood. In adolescence, scores were derived by summing up the items within each SDQ subscale, as commonly applied with this instrument, and subsequently normalizing the resulting scores to a 0–1 scale. In young adulthood, items from the PHQ‐4, DSM‐5‐CC, and PID‐5 were first Min‐Max normalized (0–) to account for differences in response scales, and then mean scores were calculated for each dimension. Given the right‐skewed distribution of the resulting scores, a logarithmic transformation was applied. As a check of internal consistency, we estimated McDonald's *ω* for the identified factors. To respect item ordinality and skew, *ω* was computed from polychoric correlation matrices based on the ordinal item responses (i.e., prior to min–max normalization and log transformation).

In the second step of the analysis, linear regression models were used to examine associations between adolescent symptoms and young adult outcomes. Internalizing and externalizing factor scores derived from the SDQ served as predictors, while factor scores from the PHQ‐4, DSM‐5‐CC, and PID‐5 represented outcomes in young adulthood. To account for outliers and heteroscedasticity, robust linear regression models using ML‐estimation were employed, ensuring stable and reliable parameter estimates less sensitive to violations of regression assumptions. For regression analyses, weighting factors were applied to correct for selective participation effects, ensuring that the weighted sample matched the distribution of participants from KiGGS Wave 2. Details on the weighting factor composition and procedures are available in Lange et al. (2025) (Lange et al. 2025).

To quantify the smallest effects detectable with our data, we conducted a post hoc sensitivity analysis for the regression models (two‐sided *α* = 0.05, 80% power). Using the model degrees of freedom (*N* = 2172; 18 predictors including covariates; residual df = 2153), we computed the minimally detectable effect size as Cohen's *f* ^2^ = 0.0036, corresponding to partial *R* ^2^ ≈ 0.0036 and partial correlation (*r* ≈ 0.06). This sensitivity analysis confirmed that the models were sufficiently powered to detect small effects.

Although the adolescent and adult factors are intended to index the same broad spectra, formal longitudinal measurement invariance (configural/metric/scalar) could not be tested because the waves used non‐overlapping item sets and response. Without identical anchor items administered at both time points, multi‐group models are unidentified and equality constraints on loadings/intercepts are not interpretable. Accordingly, we treat the longitudinal analyses as predictive links between conceptually matched constructs, not as tests of latent mean change.

To quantify the smallest effects detectable with our data, we conducted a post hoc sensitivity analysis for the multiple‐regression models (two‐sided *α* = 0.05, 80% power). Using the model degrees of freedom (*N* = 2172; 18 predictors including covariates; residual df = 2153), we computed the minimally detectable effect size as Cohen's f^2^ and reported its equivalents as partial *R* ^2^ (=f^2^/[1 + f^2^]) and the corresponding partial correlation (partial *R* ^2^).

All analyses were conducted in R (version 4.4.1). Confirmatory factor analyses (CFA) were performed using the lavaan package (Rosseel 2012), exploratory factor analyses (EFA) using the psych package (Revelle 2021), and robust linear regression models using the MASS package (rlm function) (Venables and Ripley 2004).

## 4 Results

### 4.1 Sample Description

The JEPSY dataset included 3051 participants aged 16–25. For longitudinal regression analyses, 2172 participants aged 18–26 years with complete data from adolescence and young adulthood were included. The mean age at follow‐up was 22 years, with 68.3% being female. Educational attainment was high, with 80.6% having completed high school or higher degree. Most participants (82.2%) rated their general health as very good or good.

In adolescence (11–17 years at KiGGS wave 2), internalizing symptoms were assessed via emotional and peer problems, while externalizing symptoms included hyperactivity/inattention and conduct problems. Mean scores (range 0–2) indicated that emotional difficulties (e.g., fears: 1.87) and peer problems (e.g., often alone: 0.68) were more common, whereas conduct problems were less frequent (e.g., steals: 0.07). Substance use behaviors in adolescence showed that 62.7% were non‐drinkers, 27.3% were moderate drinkers, and 10.1% were at‐risk drinkers. Smoking was rare, with 97.7% reporting no regular smoking (see Table 1).

Adult internalizing symptoms were captured via the PHQ‐4 and DSM‐5‐CC, while externalizing personality traits were assessed using the Personality Inventory for DSM‐5 (PID‐5). Based on the PHQ‐4, anxiety symptoms were more prevalent than depressive symptoms, with mean anxiety scores ranging from 0.94 to 1.13, compared to 0.75 to 0.89 for depressive symptoms. Substance use scores (range 0–4) remained low on average, with alcohol use at 0.83, tobacco use at 0.79, and non‐prescription drug use at 0.13. Externalizing personality traits, including risk‐taking and impulsivity, had mean scores of 0.69 and 0.80, respectively. Detailed descriptive statistics for all measures are provided in Table 1.

### 4.2 Factor Analysis

#### 4.2.1 Adolescence (11–17 years)

A CFA was conducted to examine how the SDQ represents internalizing and externalizing symptom spectra in adolescence based on prior evidence(Goodman 2001).

The initial two‐factor model, which grouped symptoms into internalizing and externalizing categories, showed poor model fit (CFI = 0.834, TLI = 0.813, RMSEA = 0.074, SRMR = 0.093), and several items (e.g., fears, fights, attentiveness) had weak loadings (Supporting Information S1). After removing poorly loading items (< 0.30), fit indices improved but still failed to meet acceptable thresholds (e.g., CFI = 0.896, TLI = 0.881, RMSEA = 0.066) (Supporting Information S1). Therefore, the two‐factor model was deemed insufficient.

A subsequent four‐factor model—distinguishing emotional problems, peer problems, conduct problems, and hyperactivity/inattention—showed better fit (CFI = 0.881, TLI = 0.863, RMSEA = 0.064, SRMR = 0.077). Further refinement by removing low‐loading items resulted in a final model with good fit (CFI = 0.960, TLI = 0.952, RMSEA = 0.041, SRMR = 0.061), supporting the four‐factor structure as the most appropriate representation of the SDQ data (Supporting Information S1).

#### 4.2.2 Young Adulthood (16–25 years)

In the EFA of young adulthood mental health outcomes—including items from the PHQ‐4, DSM‐5‐CC, and PID‐5—an initial four‐factor solution emerged based on eigenvalues greater than 1. However, the item related to detachment (DSM‐5‐CC) initially loaded onto a unique factor, separate from the internalizing spectrum, which included anxiety and depression symptoms. This is consistent with the HiTOP model, where detachment is recognized as an independent construct, distinct from core internalizing symptoms.

After excluding the detachment item, the three‐factor solution provided the best conceptual fit and aligned well with the HiTOP framework. This three‐factor solution comprised: (1) an internalizing factor, with PHQ‐4 and DSM‐5 items for anxiety and depressive symptoms, such as nervousness, and anhedonia; (2) a substance use factor, with DSM‐5‐CC items for alcohol, tobacco, and non‐prescription drug use; and (3) an externalizing personality trait factor, which encompassed PID‐5 items related to disinhibition, including risk‐taking and impulsivity. These findings supported a distinct separation between internalizing symptoms and two externalizing components—substance use and personality traits—in alignment with the HiTOP model. Results of the final EFA model are presented in Supporting Information S1.

A subsequent CFA confirmed the three‐factor structure (CFI = 0.985, TLI = 0.981, RMSEA = 0.055, SRMR = 0.054), with all item loadings statistically significant (*p* < 0.001) on their respective latent factor (Supporting Information S1). Cross‐validation using a population‐representative dataset also supported the three‐factor solution with good model fit (CFI = 0.994, RMSEA = 0.042) (Supporting Information S1).

Internal consistency was acceptable to excellent for the young‐adult composites: substance use *ω* = 0.74, externalizing traits *ω* = 0.75, internalizing *ω* = 0.94. These values are in line with expectations given the short length of the substance‐use/externalizing scales and the broader coverage of the internalizing spectrum.

Descriptive statistics for the standardized factor scores are summarized in Supporting Information S1.

### 4.3 Results of Regression Analyses

Table 2 summarizes the multiple linear regression models predicting internalizing and externalizing symptoms in young adulthood.

**TABLE 2:** Multiple linear regression models predicting internalizing and externalizing symptoms and substance use in young adulthood (*n* = 2172).

Predictor					Externalizing							
	Internalizing				Personality traits				Substance use			
	Std. Est.	Std. Error	*p*	95% CI	Std. Est.	Std. Error	*p*	95% CI	Std. Est.	Std. Error	*p*	95% CI
Adolescent predictors												
Emotional problems	**0.171**	0.023	< 0.001	0.13–0.22	0.033	0.025	0.189	−0.02–0.08	0.034	0.023	0.145	−0.01–0.08
Peer problems	**0.082**	0.021	< 0.001	0.04–0.12	**0.062**	0.024	0.010	0.02–0.11	**−0.078**	0.021	< 0.001	−0.12 to −0.04
Hyperactivity	0.034	0.021	0.113	−0.01–0.08	**0.144**	0.023	< 0.001	0.10–0.19	**0.067**	0.024	0.002	0.03−0.11
Conduct problems	0.033	0.024	0.178	−0.01–0.08	**0.167**	0.026	< 0.001	0.12–0.22	0.014	0.026	0.381	−0.02–0.07
Drinking (Ref. Non‐drinker)												
Moderate	−0.029	0.053	0.589	−0.14–0.07	0.030	0.058	0.602	−0.08–0.14	**0.012**	0.054	< 0.001	0.12–0.33
Risky	−0.091	0.073	0.210	−0.24–0.05	0.102	0.081	0.212	−0.06–0.26	**0.021**	0.075	< 0.001	0.25–0.55
Regular smoking (Ref. No)												
Yes	−0.005	0.019	0.795	−0.05–0.03	**0.069**	0.022	0.002	0.03–0.11	**0.059**	0.028	< 0.001	0.12–0.23
Parent socioeconomic status												
Occupation	0.022	0.026	0.398	−0.03–0.07	0.030	0.028	0.284	−0.03–0.09	0.000	0.026	0.972	−0.05–0.05
Education	0.017	0.025	0.501	−0.03–0.07	0.009	0.027	0.724	−0.04–0.06	0.001	0.025	0.535	−0.03–0.07
Income	−0.003	0.022	0.210	−0.05–0.04	−0.033	0.025	0.181	−0.08–0.02	0.001	0.022	0.281	−0.02–0.07
Young adult predictors												
Sex (Ref. Male)	**0.086**	0.019	< 0.001	0.05–0.13	**−0.123**	0.022	< 0.001	−0.17 to −0.08	**−0.119**	0.021	< 0.001	−0.16 to −0.08
Age	−0.028	0.027	0.293	−0.08–0.03	**−0.075**	0.028	0.007	−0.13 to −0.02	**−0.080**	0.026	0.002	0.13 to −0.03
Education (Ref. No/low)												
Middle	0.279	0.186	0.133	−0.09–0.66	0.021	0.193	0.913	−0.38–0.41	−0.007	0.408	0.752	−0.94–0.67
High	0.290	0.184	0.115	−0.08–0.65	−0.056	0.190	0.769	−0.45–0.32	−0.019	0.407	0.363	−1.18–0.43
In education	**0.728**	0.251	0.004	0.22–1.20	0.242	0.286	0.396	−0.34–0.80	−0.016	0.433	0.479	−1.18–0.54
Subjective social status	**−0.139**	0.020	< 0.001	−0.18 to −0.11	−0.037	0.022	0.101	−0.81–0.01	**−0.002**	0.021	0.021	−0.09–0.01
Household composition (Ref. Living alone)												
Not living alone	−0.015	0.019	0.418	−0.05–0.02	−0.034	0.021	0.111	−0.08–0.01	−0.004	0.019	0.104	−0.07–0.01
Self‐perceived health (Ref. Very good/good)												
Fair/Bad/Very bad	**1.017**	0.056	< 0.001	0.91–1.13	**0.424**	0.065	< 0.001	0.30–0.55	**0.261**	0.069	< 0.001	0.13–0.39

*Note:* Significant results (*p* < 0.05) are highlighted in bold.

Abbreviations: CI = Confidence Interval; Std. Est. = Standardized Estimate, Std. Error = Standard Error, Ref = Reference Category.

#### 4.3.1 Predicting Internalizing Symptoms

Adolescent emotional (*β* = 0.171, *p* < 0.001) and peer problems (*β* = 0.082, *p* < 0.001) were positively associated with internalizing symptoms in young adulthood. Externalizing symptoms (hyperactivity, conduct problems) and adolescent substance use showed no significant effects.

In young adulthood, female sex (*β* = 0.086, *p* < 0.001) and self‐perceived bad or very bad health (fair: *β* = 0.939, *p* < 0.001; bad/very bad: *β* = 1.691, *p* < 0.001) were significant predictors of higher internalizing symptoms. Additionally, subjective social status (*β* = −0.139, *p* < 0.001) was negatively associated with internalizing symptoms, indicating that individuals with a lower perceived social status reported higher symptom severity. Neither age nor education levels had a significant effect.

#### 4.3.2 Predicting Externalizing Symptoms

Hyperactivity (*β* = 0.144, *p* < 0.001) and conduct problems (*β* = 0.167, *p* < 0.001) in adolescence predicted externalizing personality traits in young adulthood. Peer problems, classified as an internalizing dimension, also showed a weaker but significant association with externalizing symptoms (*β* = 0.062, *p* = 0.010). Among substance use behaviors, childhood smoking emerged as a significant predictor (*β* = 0.069, *p* = 0.002), while childhood alcohol use did not show a significant association.

In young adulthood, age (*β* = −0.075, *p* = 0.007) was negatively associated with externalizing symptoms, suggesting fewer symptoms with increasing age. Poorer self‐rated health in adulthood was linked to higher externalizing symptoms (fair: *β* = 0.407, *p* < 0.001; bad/very bad: *β* = 0.544, *p* = 0.001). Female sex was associated with lower externalizing symptom levels (*β* = −0.123, *p* < 0.001).

#### 4.3.3 Predicting Substance Use

Hyperactivity (*β* = 0.067, *p* = 0.002) in adolescence predicted higher substance use in adulthood, while peer problems (*β* = −0.078, *p* < 0.001) were negatively associated, suggesting that adolescents with social difficulties were less likely to engage in later substance use. Smoking (*β* = 0.059, *p* < 0.001) and both moderate (*β* = 0.012, *p* < 0.001) and risky drinking (*β* = 0.021, *p* < 0.001) in adolescence were significant predictors for more frequent/intense substance use.

In adulthood, lower subjective social status (*β* = −0.037, *p* = 0.002), poorer self‐rated health (fair: *β* = 0.407, *p* < 0.001; bad/very bad: *β* = 0.544, *p* = 0.001), and younger age (*β* = −0.080, *p* = 0.002) were associated with higher substance use. Female sex was linked to lower levels of substance use (*β* = −0.119, *p* < 0.001).

Sensitivity analysis shows that the models were powered to detect small effects. With *N* = 2172 and 18 predictors, the minimally detectable effect at 80% power (*α* = 0.05) was *f* ^2^ = 0.0036, corresponding to partial *R* ^2^ ≈ 0.0036 and partial *r* ≈ 0.060.

**FIGURE 1:**
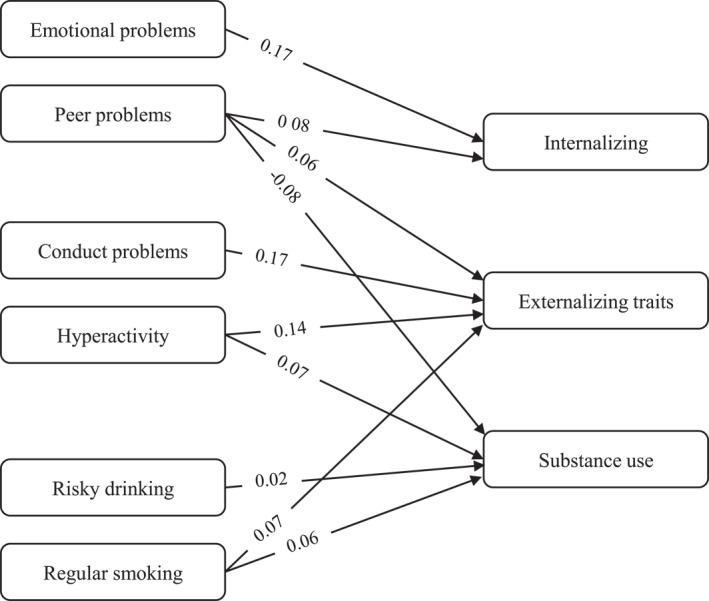
Predictive pathways from adolescent SDQ dimensions to young‐adult spectra (JEPSY). Only significant standardized paths (*β*, *p* < 0.05) are displayed. Models are robust and weighted; paths adjusted for age, sex, and SES. Non‐significant paths were excluded for readability.

Figure 1 illustrates the significant cross‐wave associations (standardized *β*, *p* < 0.05), with non‐significant paths suppressed for readability.

## 5 Discussion

This study investigated the factorial structure, continuity, and comparability of internalizing and externalizing symptom spectra, thereby addressing critical gaps in monitoring and comparing psychopathology across the transition from adolescence to adulthood in a population‐based cohort. Our findings suggest that broad, transdiagnostic dimensions can be reliably captured over time, even when different assessment instruments are employed, reinforcing the utility of the HiTOP framework for mapping the continuity of psychopathological symptom spectra.

Factor analyses of adolescent SDQ data revealed that a two‐factor model (internalizing vs. externalizing) provided poor model fit, with specific items (e.g., attentiveness, fears) exhibiting weak loadings, and suggesting that adolescent psychopathology cannot be fully captured by a binary classification. In contrast, a refined four‐factor model demonstrated superior fit, aligning with previous research (Goodman 2001; Hawes and Dadds 2004; He et al. 2013). This underscores the importance of refining measurement models, for instance, by excluding items with weak factor loadings (e.g., attentiveness), which have been shown to load onto minor factors (Karlsson et al. 2022). However, other studies have found support for a higher‐order structure in which internalizing and externalizing symptoms load onto two overarching factors (Dickey and Blumberg 2004). These varying findings highlight the complexity of the SDQ's factor structure and the need for ongoing research to clarify its optimal dimensional model in diverse contexts.

In young adulthood, the EFA identified three distinct dimensions: internalizing symptoms, externalizing personality traits, and substance use. Notably, the externalizing personality traits factor encompassed both disinhibited characteristics (e.g., impulsivity, risk‐taking, irresponsibility) and antagonistic traits (e.g., deceitfulness), while substance use formed a separate subdimension, aligning with the HiTOP framework (Kotov et al. 2017; Krueger et al. 2021).

Regression analyses further demonstrated the longitudinal predictive validity of these dimensions. Adolescent internalizing problems, specifically emotional and peer relationship problems, were significant predictors of internalizing symptoms in young adulthood. In contrast, hyperactivity and conduct problems, which reflect externalizing behaviors, predicted externalizing personality traits and substance use in adulthood.

The combination of stable broad spectra across 7–10 years and the identified longitudinal links is consistent with a hierarchical account in which a general liability to psychopathology (*p*) sustains covariation across domains while spectrum‐specific variance differentiates pathways (Caspi et al. 2024). The observed split of externalizing in young adulthood into disinhibitory/antagonistic traits versus substance use mirrors HiTOP predictions that externalizing comprises separable liabilities (Kotov et al. 2017; Krueger et al. 2021). Consistent with our findings, developmental work indicates that *p* is detectable early and remains relatively stable through late childhood and adolescence (Murray et al. 2016). Recent adolescent findings that link *p* to maladaptive personality features also converge with our observation of a distinct externalizing trait component in young adulthood (Benzi et al. 2025, 2024).

Further, our findings suggest distinct long‐term associations between adolescent and young adult externalizing symptom patterns. While both hyperactivity and conduct problems in adolescence were linked to externalizing personality traits in young adulthood, only hyperactivity significantly predicted later substance use. This supports the notion that substance use and other externalizing behaviors may be driven by distinct mechanisms. Impaired reward processing, for example, has been implicated as one mechanism explaining substance use by reducing sensitivity to natural rewards, increasing reliance on substances as compensation (Koob and Le Moal 2008). In contrast, behaviors such as rule‐breaking and proactive aggression, which are characteristic of antagonistic traits, are more closely linked to socio‐emotional explanatory mechanisms such as deficits in affective empathy and a diminished sensitivity to social consequences (Blair 2013). These findings highlight that while both forms of externalizing behaviors share common risk factors, their underlying psychological mechanisms may differ, influencing their long‐term trajectories in unique ways.

Additionally, the continuity observed in symptom spectra across developmental stages may provide valuable insights for early detection of mental health issues in adolescence. This understanding could be instrumental in the development of transdiagnostic screening approaches that identify risk factors for a range of disorders at an earlier stage. Such early identification could lead to more targeted interventions and potentially prevent the development of more severe psychopathological symptoms in adulthood.

Notably, peer relationship problems in adolescence were negatively associated with substance use in adulthood. This suggests that social problems may limit exposure to peer networks where risky behaviors typically emerge. Prior research has shown that greater social integration during adolescence is associated with increased substance use, as it facilitates exposure to peer modeling and access to substances (Moody et al. 2011). According to social learning theory, adolescents are particularly susceptible to adopting behaviors observed in their peer environment (Trucco 2020). Thus, a lack of peer involvement may, in some cases, reduce opportunities for engagement in substance use (Allen et al. 2005; Gamache et al. 2022).

Given the low prevalence of adolescent substance use in our sample, post‐hoc analyses examined age differences (< 14 vs. ≥ 14 years). Older adolescents were more likely to drink and smoke (*p* < 0.001), but age did not significantly moderate the association between adolescent and adult substance use.

By providing a standardized framework for mapping symptoms across adolescence and the transition to adulthood, the identified factors offer significant potential for future longitudinal and population‐based research. They can be applied in population‐based studies to yield estimates of psychological burden that are independent of specific diagnostic thresholds, thereby enabling cross‐cohort comparisons and facilitating a more comprehensive understanding of transdiagnostic mental health (problem) and comorbidity patterns (Lahey et al. 2014). Thereby, these factors serve as a foundation for investigating transdiagnostic mechanisms—such as coping strategies and resilience—that may mediate the development and persistence of psychopathological symptoms (Panagou and MacBeth 2022).

Although standardized coefficients are modest at the individual level, their population implications are non‐trivial. Assuming approximate normality, a *β* = 0.171 for adolescent emotional problems predicting adult internalizing corresponds to an expected shift from the 50th to about the 57th percentile; for externalizing traits, *β* = 0.144 (hyperactivity) and *β* = 0.167 (conduct problems) map to shifts from the 50th to roughly the 56th–57th percentile; for substance use, *β* = 0.067 (hyperactivity) maps to 53rd percentile, with early smoking/risky drinking adding small further increments. At the population level, such mean shifts change how many individuals cross high‐symptom cutoffs: e.g., a 0.17 SD shift increases the proportion above a top‐quintile threshold from about 20% to 25% and above a top‐decile threshold from 10% to 13%. These patterns support investment in universal and indicated prevention that targets shared vulnerabilities across spectra rather than focusing solely on discrete disorders. School‐ and community‐based interventions that foster adaptive emotional, social, and cognitive abilities—such as emotional regulation and social connectedness—while addressing early risk factors (e.g., impulsivity, peer difficulties) may be particularly effective in promoting long‐term mental well‐being.

Methodologically, our harmonization shows that spectrum‐level constructs can be recovered across development without identical instruments when the targets are pre‐specified (here via the HiTOP spectra), the latent structure is estimated within each wave, scores are placed on comparable scales with simple, transparent transformations, and the resulting structure is cross‐validated in an independent dataset. Combined with design weights and robust regression, this yielded coherent longitudinal links despite differences in measures. Looking ahead, large panels can plan harmonization from the outset by retaining a small anchor core of identical items per spectrum across waves to stabilize factor solutions and comparability, and inserting short bridge modules when instruments change to create clean links between scales.

Future research should also extend findings of this study by testing whether similar spectrum structures and longitudinal links hold in clinical samples and diverse cultural contexts, by broadening coverage to underrepresented parts of internalizing and externalizing, and by embedding planned anchors to permit formal assessment of longitudinal comparability. Such efforts will improve our understanding of mental health trajectories and inform tailored public health strategies to support mental well‐being across the lifespan.

### 5.1 Limitations

Several limitations should be considered when interpreting the findings of this study. First, the cohort was predominantly female (68.3%), which may limit generalizability. Although sex was included as a covariate, future work should test sex‐specific pathways explicitly. Second, although we harmonized adolescent and adult assessments using latent variable methods, residual non‐equivalence across instruments and informants cannot be fully excluded; prospective designs with planned anchor items would further strengthen longitudinal comparability. However, this approach aligns with recent recommendations for data harmonization in large‐scale mental health research, which emphasize the use of latent variable models to improve measurement consistency across instruments, time points, and diverse populations (Neidhart et al. 2024). Nonetheless, future research should aim to develop instruments that provide consistent measurements across age groups to further improve longitudinal comparability. In addition, severely affected and/or clinical populations were not sufficiently represented in the present population‐based sample. Further research should assess whether similar factor structures emerge in clinical groups or populations from diverse cultural backgrounds and across the adult lifespan. Our study focused on broader measures that assess major sections of HiTOP. We also note that many reliable and valid instruments have been developed to assess specific aspects of the nosology. These include measures assessing multiple symptom or trait dimensions within posttraumatic stress disorder (Gootzeit et al. 2015; Weathers et al. 1993), specific phobia (Cutshall and Watson 2004) and sleep disorders (Koffel and Watson 2010). Additionally, not all symptoms or components of the internalizing and externalizing spectra were assessed in the current study. For instance, distress and eating pathology within the internalizing spectrum were not included. Future research should explore the role these unexamined dimensions might play in shaping the overarching spectra and whether they integrate into the observed factor structure, or form distinct factors. Finally, while our research team spans psychology, epidemiology, and psychiatry, additional perspectives (e.g., developmental neuroscience, sociology, qualitative inquiry, and network modeling) could offer complementary insights and further refine measurement choices and interpretations.

### 5.2 Conclusion

This study highlights the importance of early identification and intervention for emotional and behavioral problems in adolescence as a means to promote mental health in adulthood. The demonstrated continuity of symptom spectra in accordance with HiTOP, despite differences in measurement tools, underscores the feasibility of harmonized approaches for studying psychopathology in population‐based settings. By building on these findings, future research should further elucidate the mechanisms underlying mental health trajectories and support the development of effective prevention and intervention strategies.

## Author Contributions

**Vera Birgel:** conceptualization, data curation, formal analysis, investigation, methodology, writing – original draft, writing – review and editing. **Michael Rapp:** conceptualization, funding acquisition, methodology, project administration, writing – review and editing. **Mira Tschorn:** conceptualization, methodology, writing – review and editing. **Heike Hölling:** conceptualization, funding acquisition, project administration, writing – review and editing. **Caroline Cohrdes:** conceptualization, funding acquisition, investigation, validation, supervision, methodology, project administration, writing – review and editing.

## Funding

The study was funded by the Federal Ministry of Research, Technology and Space (Bundesministerium für Bildung und Forschung [BMBF]) and within the initial phase of the German Center for Mental Health (DZPG) (grant: 01EE2301 F).

## Ethics Statement

All participants gave their informed consent before participating in this study. The study (application number 2024‐03‐14‐WV) was positively evaluated by the institute's data protection officer and the Ethics Committee of the German Psychological Society [DGPs] and was conducted in accordance with the latest version of the Declaration of Helsinki.

## Conflicts of Interest

The authors declare no conflicts of interest.

## Supporting information


Supporting Information S1

Supporting Information S2

## Data Availability

A scientific use file of the KiGGS survey data is available upon request. The JEPSY data can be made available on request to the Secure Data Center at the Robert Koch Institute for non commercial research. Requests should be directed to fdz@rki.de.
