# Supplementary material for: Development and Comparability of Internalizing and Externalizing Symptom Spectra From Adolescence to Young Adulthood
Source: Int J Methods Psychiatr Res. 2026 Jan 21;35(1):e70055. doi: 10.1002/mpr.70055 (PMC12820720; doi:10.1002/mpr.70055)
Supplement: Supplementary file 1 — Supporting Information S1 [file MPR-35-e70055-s001.docx]

Table S1 Standardized Factor Loadings for the SDQ Two-Factor Model (Initial CFA), age 11-18 (n = 2,234)

| Item no |  | Internalizing | Externalizing |
| --- | --- | --- | --- |
| 3  8  13  16  24  6  11*  14*  19  23 | Somatic  Worries  Unhappy  Nervous  Fears  Often alone  Prefers adults  Good friend  Popular  Bullied | 0.480  0.632  0.807  0.516  0.207  0.467  0.579  0.519  0.632  0.461 |  |
| 5  7*  12  18  22  2  10  15  21*  25* | Tempers  Obedient  Fights  Lies  Steals  Restless  Fidgety  Distractible  Reflective  Attentive |  | 0.563  0.405  0.380  0.497  0.434  0.785  0.654  0.674  0.501  0.075 |
| CFI = 0.834, TLI = 0.813, RMSEA = 0.074, SRMR = 0.093 | | | |

All factor loadings are statistically significant at p<0.001

^*^Reverse coded items were recoded

Table S2 Standardized Factor Loadings for the SDQ Two-Factor Model (CFA after Removal of Items), age 11-18 (n = 2,234)

| Item no |  | Internalizing | Externalizing |
| --- | --- | --- | --- |
| 3  8  13  16  6  11*  14*  19  23 | Somatic  Worries  Unhappy  Nervous  Often alone  Prefers adults  Good friend  Popular  Bullied | 0.480  0.626  0.808  0.515  0.467  0.574  0.515  0.631  0.457 |  |
| 5  7*  18  22  2  10  15  21* | Tempers  Obedient  Lies  Steals  Restless  Fidgety  Distractible  Reflective |  | 0.560  0.406  0.497  0.419  0.789  0.659  0.678  0.500 |
| CFI = 0.896, TLI = 0.881, RMSEA = 0.066, SRMR = 0.088 | | | |

All factor loadings are statistically significant at p<0.001

^*^Reverse coded items were recoded

Table S3 Standardized Factor Loadings for the SDQ Four-Factor Model, age 11-18 (Initial CFA) (n = 2,234)

| Item no. |  | Emotional problems | Conduct problems | Peer problems | Hyperactivity |
| --- | --- | --- | --- | --- | --- |
| 3  8  13  16  24 | Somatic  Worries  Unhappy  Nervous  Fears | 0.526  0.680  0.888  0.552  0.228 |  |  |  |
| 6  11*  14*  19  23 | Often alone  Prefers adults  Good friend  Popular  Bullied |  | 0.544  0.684  0.608  0.725  0.535 |  |  |
| 5  7*  12  18  22 | Tempers  Obedient  Fights  Lies  Steals |  |  | 0.627  0.381  0.416  0.539  0.451 |  |
| 2  10  15  21*  25* | Restless  Fidgety  Distractible  Reflective  Attentive |  |  |  | 0.820  0.688  0.691  0.516  0.091 |
| CFI = 0.879, TLI = 0.860, RMSEA = 0.064, SRMR = 0.077 | | | | | |

All factor loadings are statistically significant at p<0.001

^*^Reverse coded items were recoded

Table S4 Standardized Factor Loadings for the SDQ Four-Factor Model, age 11-18 (CFA after Removal of Items) (n = 2,234)

| Item no. |  | Emotional problems | Peer problems | Conduct problems | | Hyperactivity |
| --- | --- | --- | --- | --- | --- | --- |
| 3  8  13  16 | Somatic  Worries  Unhappy  Nervous | 0.521  0.676  0.884  0.556 |  |  | |  |
| 6  11*  14*  19  23 | Often alone  Prefers adults  Good friend  Popular  Bullied |  | 0.551  0.686  0.608  0.718  0.531 |  |  | |
| 5  12  18  22 | Tempers  Fights  Lies  Steals |  |  | 0.640  0.416  0.550  0.450 |  | |
| 2  10  15  21* | Restless  Fidgety  Distractible  Reflective |  |  |  | 0.837  0.695  0.681  0.481 | |
| CFI = 0.960, TLI = 0.952, RMSEA = 0.041, SRMR = 0.061 | | | | | | |

All factor loadings are statistically significant at p<0.001

^*^Reverse coded items were recoded

Table S5 Final EFA Loadings for Young Adulthood, age 16-26 (n = 3,048)

| Item | Factor 1 (Internalizing) | Factor 2 (Externalizing, Substance Use) | Factor 3 (Externalizing, Personality Traits) |
| --- | --- | --- | --- |
| Alcohol use  Tabacco use  Prescription drug use  Non-prescription drug use |  | 0.54  0.77  0.63  0.29 |  |
| Risk Taking  Irresponsibility  Eccentricity  Deceitfulness  Impulsivity |  |  | 0.58  0.58  0.48  0.46  0.57 |
| Nervous (DSM5-CC)  Panic (DSM5-CC)  Avoiding situations (DSM5-CC)  PHQ1  PHQ2  GAD1  GAD2  Sleep problems (DSM5-CC)  Anhedonia (DSM5-CC)  Social anhedonia (DSM5-CC) | 0.78  0.79  0.67  0.66  0.78  0.73  0.77  0.48  0.73  0.68 |  |  |

All factor loadings are statistically significant at p<0.001

Table S6 Final CFA Loadings for Young Adulthood, age 16-26 (n = 3,048)

| Item | Factor 1 (Internalizing) | Factor 2 (Externalizing, Substance Use) | Factor 3 (Externalizing, Personality Traits) |
| --- | --- | --- | --- |
| Alcohol use  Tabacco use  Prescription drug use  Non-prescription drug use |  | 0.321  0.700  0.792  0.621 |  |
| Risk Taking  Irresponsibility  Eccentricity  Deceitfulness  Impulsivity |  |  | 0.481  0.521  0.623  0.486  0.694 |
| Nervous (DSM5-CC)  Panic (DSM5-CC)  Avoiding situations (DSM5-CC)  PHQ1  PHQ2  GAD1  GAD2  Sleep problems (DSM5-CC)  Anhedonia (DSM5-CC)  Social anhedonia (DSM5-CC) | 0.787  0.800  0.647  0.774  0.792  0.720  0.777  0.501  0.869  0.769 |  |  |
| CFI = 0.985, TLI = 0.981, RMSEA = 0.055, SRMR = 0.054 | | | |
| All factor loadings are statistically significant at p<0.001 | | | |

Table S7 Cross-Validation of CFA via MDS data, age 16-25 (n = 232)

| Item | Factor 1 (Internalizing) | Factor 2 (Externalizing, Substance Use) | Factor 3 (Externalizing, Personality Traits) |
| --- | --- | --- | --- |
| Alcohol use  Tabacco use  Prescription drug use  Non-prescription drug use |  | 0.371  0.763  0.835  0.546 |  |
| Risk Taking  Irresponsibility  Eccentricity  Deceitfulness  Impulsivity |  |  | 0.527  0.767  0.659  0.559  0.747 |
| Nervous (DSM5-CC)  Panic (DSM5-CC)  Avoiding situations (DSM5-CC)  PHQ1  PHQ2  GAD1  GAD2  Sleep problems (DSM5-CC)  Anhedonia (DSM5-CC)  Social anhedonia (DSM5-CC) | 0.804  0.842  0.646  0.827  0.879  0.714  0.827  0.608  0.858  0.817 |  |  |
| CFI = 0.994, TLI = 0.993, RMSEA = 0.042, SRMR = 0.070 | | | |

All factor loadings are statistically significant at p<0.001

Table S8 Descriptive Statistics for Calculated Factor Scores (n = 2,172)

| Factor | M | SD | Skewness |
| --- | --- | --- | --- |
| **Adolescent Factors** |  |  |  |
| Hyperactivity | 0.28 | 0.25 | 0.70 |
| Conduct Problems | 0.11 | 0.14 | 1.51 |
| Emotional Problems | 0.29 | 0.23 | 0.78 |
| Peer Problems | 0.22 | 0.18 | 1.08 |
| **Young Adulthood Factors** |  |  |  |
| Substance Use | 0.13 | 0.14 | 1.47 |
| Externalizing Traits | 0.23 | 0.16 | 0.73 |
| Internalizing Symptoms | 0.30 | 0.20 | 0.90 |
