# Supplementary material for: Development and Comparability of Internalizing and Externalizing Symptom Spectra From Adolescence to Young Adulthood
Source: Int J Methods Psychiatr Res. 2026 Jan 21;35(1):e70055. doi: 10.1002/mpr.70055 (PMC12820720; doi:10.1002/mpr.70055)
Supplement: Supplementary file 2 — Supporting Information S2 [file MPR-35-e70055-s002.docx]

**Appendix I: Description of the Representative MDS Sample**

The present study utilizes data from the representative Minimum Dataset (MDS) Validation Study, an initiative embedded within the German Center for Mental Health (DZPG). The MDS aims to establish a psychometrically sound and standardized assessment framework for mental health research, balancing comprehensiveness with feasibility.

**Participants and Data Collection**

The representative sample was recruited through Respondi, a market research institute, ensuring quota-based representativeness in terms of gender (50:50 distribution), age groups (18–69 years), and self-reported general health status. A total of 2,297 participants were included in this subsample. Participants were invited via an online panel, with a response rate of 15% (10,951 responses from 74,931 invitations). Data collection took place between February 23 and March 29, 2024.

**Ethics and Inclusion Criteria**

Inclusion criteria required membership in the Respondi panel and consent to anonymous participation. No exclusion criteria were applied. The study was approved by the Ethics Committee of the University of Potsdam (Nr. 6/2016 Addendum MDS-Valid) and conducted in accordance with the Declaration of Helsinki.

**Measures**

Data collection was based on a psychometric assessment battery derived through a Delphi consensus process, ensuring the selection of core constructs relevant to both clinical and non-clinical settings. The questionnaire covered four major domains:

- Research Domain Criteria (RDoC): Positive and Negative Valence Systems, Cognitive Systems, Social Processes, Sensorimotor Systems, and Arousal/Regulatory Systems.
- Global Health: Mental and physical health status, functional limitations, and subjective well-being.
- Hierarchical Taxonomy of Psychopathology (HiTOP): Dimensional assessment of internalizing and externalizing spectra, including symptom dimensions related to depression, anxiety, disinhibited and antagonistic externalizing, and thought disorder symptoms.
- Psychometrics: Variables including trauma, depression, anxiety, stress, substance use, and social factors (e.g., loneliness, social support)

The questionnaire was designed for administration via PC, laptop, tablet, and mobile devices, ensuring broad accessibility.
